# Determining the Cytotoxicity of Oxidized Lipids in Cultured Caco-2 Cells Using Bioimaging Techniques

**DOI:** 10.3390/molecules25071693

**Published:** 2020-04-07

**Authors:** Rabia Alghazeer, Abdullah A. Burwaiss, Nazlin K. Howell, Wafa S. Alansari, Ghalia Shamlan, Areej A. Eskandrani

**Affiliations:** 1 Chemistry Department, Faculty of Science, University of Tripoli, Tripoli 50676, Libya; 2 Medicine Department, Faculty of Human Medicine, University of Tripoli, Tripoli 50676, Libya; A.burwaiss@uot.edu.ly; 3 School of Biomedical and Molecular Sciences, University of Surrey, Guildford, Surrey GU2 7XH, UK; n.howell@surrey.ac.uk; 4 Biochemistry Department, Faculty of Science, University of Jeddah, Jeddah 21577, Saudi Arabia; wsalansari@uj.edu.sa; 5 Department of Food Science and Nutrition, College of Food and agriculture Sciences, King Saud University, Riyadh 11362, Saudi Arabia; shamlana@ksu.edu.sa; 6 Chemistry Department, Faculty of Science, Taibah University, Medina 30002, Saudi Arabia; aeskandrani@taibahu.edu.sa

**Keywords:** fish oil, lipid peroxidation, ESR spectroscopy, AFM, Raman microscopy

## Abstract

Fish lipids are comprised of considerable quantities of polyunsaturated acids and are prone to oxidation, producing reactive oxygen species and hydroperoxides. This study aimed to evaluate the biochemical and structural alterations in Caco-2 cells following exposure to 100 μg/mL methyl linoleate or fish oil, and then radiated for 24, 48 or 72 h. Electron spin resonance spectroscopy detected free radicals in the lipid membrane, Raman microscopy observed biochemical alterations and atomic force microscopy identified changes in morphology, such as the breakdown of DNA bonds. The study showed that bioimaging and biochemical techniques can be effective at detecting and diagnosing cellular injuries incurred by lipid peroxidation.

## 1. Introduction

Epidemiological studies suggest polyunsaturated fatty acids (PUFAs) may be protective against colorectal neoplasia, especially those extracted from fish lipids [1]. However, these fatty acids are also known to generate lipid peroxides, which accelerate the oxidation of proteins and contribute to the development of many degenerative diseases, such as diabetes, atherosclerosis and cancer [2,3,4]. Mechanistically speaking, research has found, through the use of electron spin resonance (ESR) spectroscopy, that the damage incurred by lipid peroxides is due to free radicals inserting themselves into lipid bilayers to compromise the integrity of cell membranes, ultimately leading to cell death. Necrosis and apoptosis, two forms of cell death, differ according to variations in their structural and biochemical properties; the former causes cells to swell and rupture [5,6], whereas the latter leads to cell shrinkage and DNA fragmentation. In addition, apoptosis is associated with stimulating cysteine aspartic acid-specific proteases recognised in form of caspases [7,8]. Notably, early investigations have found PUFAs to promote apoptosis and prevent cellular proliferation in vitro [9,10].

Traditionally, cellular toxicity has been assessed by various techniques, such as fluorescence microscopy and electrophoresis [11,12]. Each experiment that employs these techniques must take into careful consideration the amount of cells and materials needed, as well as logistics pertaining to the staining procedure. Additionally, the entire process can be rather time-intensive and still fail to deliver desired results, sometimes taking up to three days to complete and not detecting anything remarkable. As such, new methods are being explored to overcome these challenges. One that has proven to be especially advantageous, in particular, is bioimaging. This requires minimal sample handling and no expensive chemicals, is efficient and can observe biological processes and compositional changes in tissues at the subcellular level. 

Examples of two bioimaging techniques that have proven quite promising, as of late, are Raman microscopy (RM) and atomic force microscopy (AFM). RM uses a laser-based microscopic device that is able to distinguish between biological compounds (e.g., nucleic acids, proteins, lipids and carbohydrates) in human cells and tissues, viruses and bacteria, by way of the intensity and frequency of their molecular vibrations [13,14,15,16,17]. It has been utilized extensively to observe cell death in cancer studies [18,19]. AFM is a scanning probe that has been used to explore microbial surfaces, producing 3D images with molecular resolution [20,21]. With a tip the size of an atom, it can measure intramolecular and intermolecular forces associated with biological systems [21,22,23,24], calculate mechanical and biological response to mechanical stimuli by measuring force–distance curves [25,26,27], and provide high-resolution images [28,29]. 

For our study, Caco-2 cells isolated from human colorectal adenocarcinoma, were used to explore the cytotoxicity of PUFAs because they have been used extensively in related nutritional, pharmacological and toxicological investigations [30]. We aimed to evaluate changes in their molecular structure and morphology using RM, AFM and ESR.

## 2. Results and Discussion

Results showed that microspectroscopy allowed direct scanning of morphological variations at the molecular level, details from the Raman spectra analysis provided valuable information on cell damage resulting from endogenous or exogenous toxic compounds. 

### 2.1. ESR Spectroscopy Images

ESR is considered as the first direct and sensitive method free radicals detecting for the study of lipid oxidation [31,32,33]. An increased production of reactive oxygen species (ROS) was detected following the oxidized lipid treatments for 4 and 24 h. Every molecule with unpaired electrons has a unique spectrum because of its nuclear spin and magnetic field. In physiological systems, ROS’s are constantly produced by mitochondria during respiration, but they are quenched by antioxidants. Excessive amounts of ROS, however, will not be fully quenched and can lead to cellular damage. 

**Figure 1:**
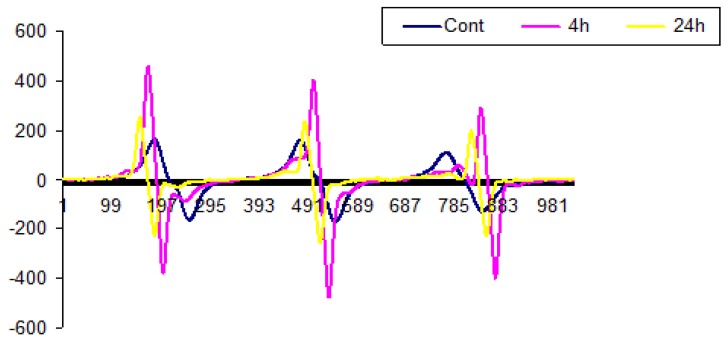
Electron spin resonance (ESR) spectra produced from Caco-2 cells incubated for 0, 4 or 24 h with 100 μg/mL (24 h) UV-radiated methyl linoleate. The spectra were recorded with a modulation of 100 kHz, 5 mW microwave power, a central magnetic field of 328.5 G and a scan of 200 and 4 G at room temperature.

ESR spectra of Caco-2 cells incubated with 24 h UV-radiated methyl linoleate for 4 or 24 h are shown in Figure 1. All samples were stored at −20 °C for one week before measurements. We previously established that free radicals are stable at −20 °C [34]. All samples showed an isotropic 3 lines spectrum with hyperfine coupling, typical of a nitroxide radical. For untreated cells, the signal had a g = 2.0053, a^N^ = 15.46. In contrast, cells incubated with 24 h UV-radiated ML for 4 h, the signal had a g = 2.0091, a^N^ = 14.76; cells with a 24 h incubation had a g = 2.0063, a^N^ = 15.76. The change in a^N^ values shows a shift in free radicals moving from lipids to protein sites [34,35]. The intensity of spectra for the oxidized ML samples was significantly higher than that of the Caco-2 cells, indicating that free radicals attacked the cell membrane and regenerated radicals. Of note, the intensity of spectra for Caco-2 cells treated with oxidized ML for 48 and 72 h was very low compared to that of the control, as well as other treatment times, so the results not shown. A decrease in the ESR signal from samples incubated with oxidized ML for 24 or 72 h, compared with 4 h incubation, suggests that free radicals are attacking other molecules and forming products, such as aldehydes [34,36].

### 2.2. AFM Images

**Figure 2:**
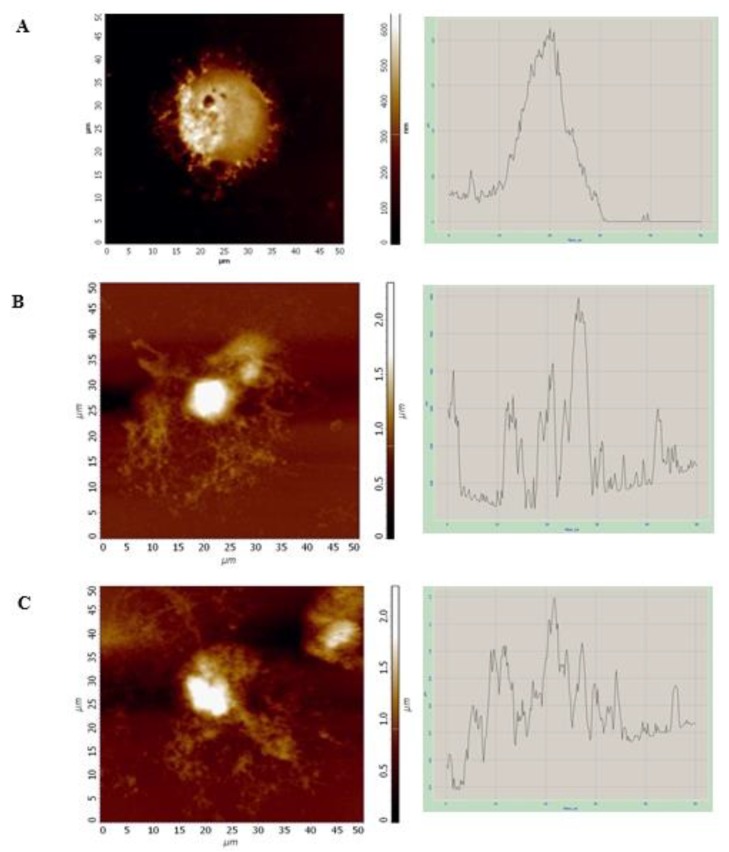
Atomic force microscopy (AFM) surface topography and line profile analysis of (**A**) untreated Caco-2 cells; (**B**) Caco-2 cells treated with 72 h UV-radiated fish oil (100 μg/mL) for 24 h; (**C**) Caco-2 cells incubated with 72 h UV-radiated ML (100 μg/mL) for 24 h.

**Figure 3:**
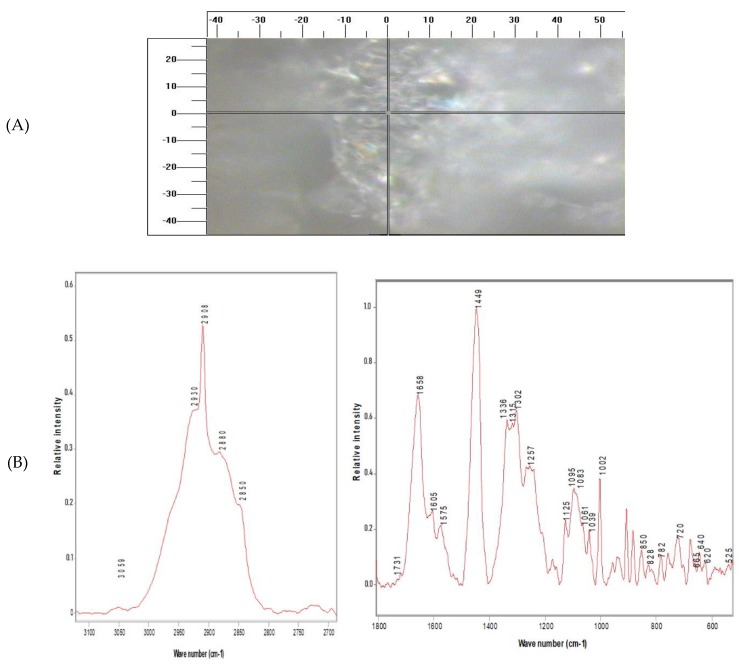
Raman microscopy (RM) of: (**A**) Caco-2 cells under 100 × objective; (**B**) Raman spectra (a) 2700–3100 cm^−1^ and (b) 1800–600 cm^−1^ regions obtained from untreated cells.

Figure 2 shows a 50 μm × 50 μm tapping-mode from topographical images of untreated and treated Caco-2 cells. The untreated cells have smooth surfaces, lacking irregularity or pores (Figure 3A), while cells incubated with 72 h UV-radiated fish oil or ML present a dramatic change in cell structure especially in the cell membrane and nucleus (Figure 3B). There was a correlation between changes in cell height and morphology: cell height increased for untreated and treated (with 72 h UV-radiated fish oil or ML) cells by 600 nm and 2000 nm, respectively, indicating disruption of the cell membrane and aggregation. A line profile analysis also showed dramatic differences between the samples: untreated cells had one peak and treated cells had many peaks, suggesting that lipid peroxidation products may be interacting with cellular components, leading to aggregation and crosslinking.

### 2.3. RM Images

Results show that information on all major cell components (lipids, proteins and nucleotides) is contained in the Raman spectra. Table 1 summarizes the assignment of wave numbers of some regions which, according to the literature, have specific biological significance [14,37,38,39,40,41]. Band at 1449 cm^−1^ was allocated to C-H deformation vibrations in cell constituents [42,43,44,45] and was used to normalize the spectra, since it hardly changes within a certain cell type. Table 2 shows the comparative intensities of the bands.

**Figure 4:**
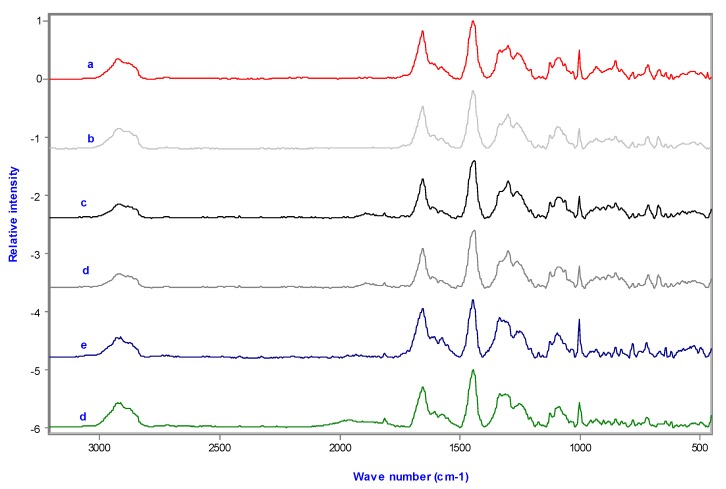
Comparison of the RM spectra (500-3500 cm^−1^) of Caco-2 cells incubated for 24 h with (100 μg/mL) of (**a**) 24, (**b**) 48 and (**c**) 72 h UV-radiated ML or (**d**) 24, (**e**) 48 and (**f**) 72 h UV-radiated fish oil.

Figure 4 shows that most of the important variations between untreated and treated cells were at locations consistent with those for nucleic acids: DNA phosphodioxy group PO^2−^ symmetric stretched at 1095 cm^−1^, DNA backbone O-P-O stretched at 788 cm^−1^ and 828 cm^−1^, vibrating DNA bases, cytosine and thymine, at 782 cm^−1^ and adenine at 728 cm^−1^. Additionally, there were differences in the C-H stretched bands at the range of 2700–3100 cm^−1^ and CH de-formatting occurred at 1450 cm^−1^; these bands were correlated with CH_3_, CH_2_ and CH functional groups.

There were also changes in the protein bands, amide I at 1660 cm^−1^, amide III in different conformations at 1238 cm^−1^, 1258 cm^−1^ (β-sheet) and 1271 cm^−1^ (α-helices), CH_2_/CH_3_ bending vibration at 1449 cm^−1^, CH_2_ twisted at 1340 cm^−1^. The CH_2_ corresponding to the lipid content in the cell membrane contributed to the band at 1302 cm^−1^ and 1449 cm^−1^ band (CH_2_ scissor mode).

**Figure 5:**
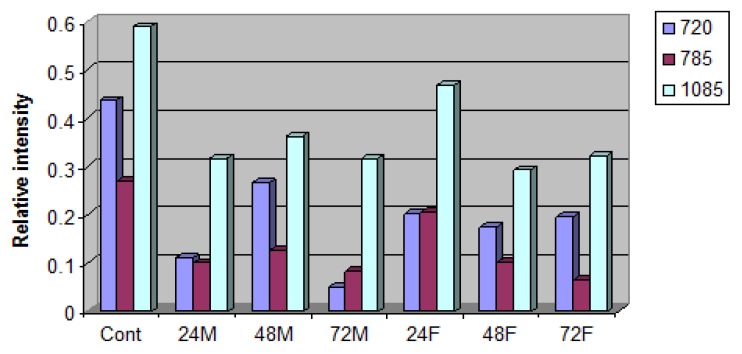
Spectral differences in Raman bands 720, 785 and 1085 cm^−1^ between freeze-dried treated and untreated cells. Caco-2 cells were incubated with 24 h ML or fish oil (F) radiated under UV light for 24, 48 and 72 h.

Figure 5 shows a reduced amount of nucleic acids in the treated cells. This reduction is indicated by a decrease in the relative intensity in the band 720 and 782 cm^−1^, corresponding to cytosine and thymine which decreased by 23%, 62% and 75% in samples treated with 24, 48 and 72 h UV-radiated fish oil, and 53%, 63% and 69% in samples treated with 24, 48 and 72 h UV-radiated ML. In addition, due to the break in the diphosphoester bonds, there was a decrease in the peak 1059 cm^−1^ by 20%, 50% and 45% in samples treated with 24, 48 and 72 h UV-radiated fish oil, and 38%, 47% and 44% in samples treated with 24, 48 and 72 h UV-radiated ML. 

**Figure 6:**
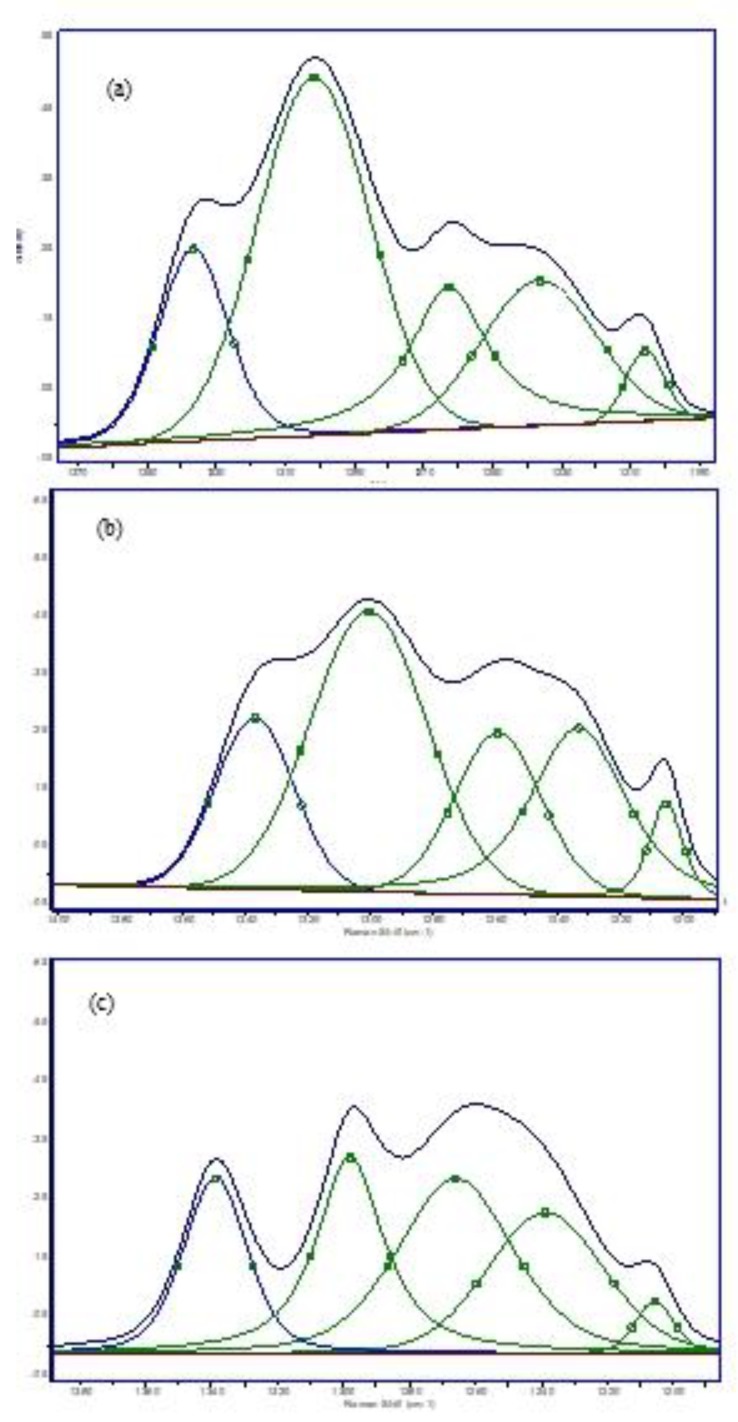
Peak fitting of 1190-1385 cm^−1^ region corresponding to (**a**) untreated Caco-2 cells and (**b**) Caco-2 cells incubated for 24 h with 72 h UV-radiated fish oil (100 μg/mL) and (**c**) ML (100 μg/mL).

A deconvolution technique was used for analysing the 1200-1400 cm^−1^ Raman region, which includes important bands pertaining to proteins, nucleic acids and lipids. The fitted peaks are the weak band 1367 cm^−1^, assigned to symmetric CH_3_-stretched phospholipids; adenine, carbohydrates, vibrations and proteins of C-H deformation are at 1342 and 1320 cm^−1^; CH_2_-twisted phospholipids are at 1301 cm^−1^; peaks at 1284 (alpha-helix), 1258 and 1242 (beta-sheets) and 1231 cm^−1^ (random coil) as well as the C-C_6_H_5_-stretched phenylanaline at 1209 cm^−1^. The in-plane deformation of =CH for lipids was donated to spectra ranging 1260 to 1300 cm^−1^. Peak fitting in the 1200-1400 cm^−1^ region indicated a significant reduction in the spectra of treated cells compared to untreated cells, especially for samples treated with 72 h UV-radiated ML or fish oil (Figure 6). The band of 1336 cm^−1^ corresponding to DNA bases adenine and guanine reduced from 38% to 21.7%, 20.4% and 17.8% (*p* < 0.03) in samples treated for 24 h with 24, 48 and 72 h UV-radiated fish oil, respectively. Similar decreases were also detected in cells incubated with oxidized ML for 24 h, decreasing by 13.6%, 21% and 18.2 % (*p* < 0.01) with 24, 48 and 72 h UV-radiated ML, respectively. Cellular membrane damage was investigated by monitoring peaks at 1231 cm^−1^, which corresponded to a random coil and thereby indicated changes in protein conformation. It has been reported that protein strands correlate with alpha-helices and beta-sheets [46,47], and in our study, this conformational change was clearly seen by an increase in the amide I band from 78 in the untreated sample to 89 and 120 in samples treated with 24 and 72 h UV-radiated ML, respectively. Furthermore, the ratio of the 1302 cm^−1^ band to the amide III band assigned to the Raman intensity of lipid vibration at 1301 cm^−1^ and Raman intensity of amide III protein band decreased from 1.7 to 1.2, confirming structural damage to the cell membrane (Table 3). Similarly, the ratio of the 1336 cm^−1^ band to the amide III band also decreased.

The Raman band at 1743 cm^−1^ has been studied by FTIR and RM and is related to non-hydrogen bonded carbonyl C=O stretching in phospholipids. The intensity of 1743 cm^−1^ peak decreased from 0.2 in the untreated sample to 0.05, 0.04 and 0.01 for samples treated with 24, 48 and 72 h UV-radiated fish oil, and 0.04, 0.07 and 0.03 in samples treated with 24, 48 and 72 h UV-radiated ML respectively, suggesting oxidative damage indicative of apoptosis [48,49]. In addition, a substantial increase in the peak at 1604 cm^−1^ was observed; and this peak increased from 0.05 in the control samples to 0.12 and 0.086 in samples incubated with 100 μg/mL 72 h UV-radiated ML and fish oil, respectively (*p* < 0.05). According to previous studies, an increase in the band of 1602 cm^−1^ is symbolic of DNA fragmentation and damage to the cellular membrane, prominent features of apoptosis [46,50].

### 2.4. PCA Findings

**Figure 7:**
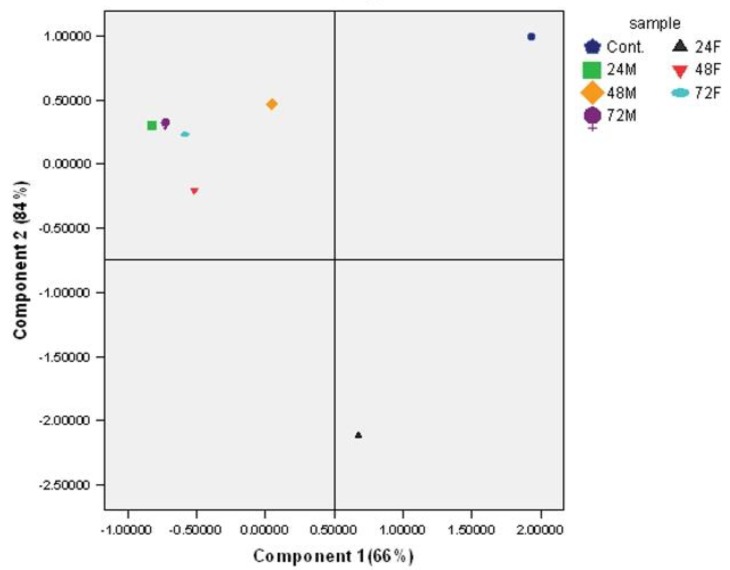
Principal component analysis (PCA) of Raman spectra of Caco-2 cells exposed to 100 μg/mL oxidized lipids for 24 h. The plot shows separation between PC1 and PC2. Treatments are related to 24, 48 and 72 h oxidized ML or fish oil (F).

Principal component analysis (PCA) is a mathematical technique for reducing the dimensionality of datasets into less variables (principal components) in order to identify the most significant variations and patterns [51,52]. Component 1 of PCA explained 66.2% of treatment variance, component 2 explained 18.1% (Table 4). The peaks assigned to proteins (1002, 1660, 1238 and 1271 cm^−1^), lipids (1449 and 1340 cm^−1^) and nucleic acids (788, 828, 782 and 1095 cm^−1^) were responsible for the differences observed between untreated and treated cells. All Raman spectral characteristics that were responsible for the separation observed in the PCA plots are shown in Table 5 [53,54]. In order to compare untreated with treated cells, PC scores were plotted where each sample was placed as distinct by Raman data (Figure 7). 

The most striking peak intensities can be seen in Table 2 for the first two components. Separation on PC1 is largely influenced by the 1129 cm^−1^ band and less by 829, 1575 and 2935 cm^−1^ Raman bands attributed to nucleic acids, proteins and lipids, indicating variation in the cell contents between the samples. Additionally, the majority of the defining peaks are in PC1.

The PC1 vs. PC2 plots for untreated and treated cells were different, presumably due to the cytotoxicity of oxidized lipids. Untreated cells and cells with 24 h UV-radiated fish oil were clearly separate from treated cells (24, 48 and 72 h UV-radiated ML, as well as 48 and 72 h UV-radiated fish oil). This separation complemented the peroxide value (PV) of oxidized lipids; thus, cells treated with 24, 48 and 72 h UV-radiated ML and 48 and 72 h UV-radiated fish oil were grouped together and had a similar PV (1000 Meq/kg), while cells treated with 24 h oxidised fish oil moved toward PC2 and had the lowest PV (200 Meq/kg). 

Cells treated with 24 and 72 h UV-radiated ML and 48 and 72 h UV-radiated fish oil were defined by high values in the C-N and C-C stretch, guanine, adenine (ring stretch) and Phe (1129, 1575 and 1606 cm^−1^), as well as low values for most of the variables assigned to DNA, lipid and proteins. Cells treated with 48 h UV-radiated ML showed a similar pattern, but to a lesser extent. Samples treated with 24 h UV-radiated fish oil were defined by a high content of DNA bands 829, 1202 and 829 cm^−1^, and a low content DNA bands 1575 and 1606 cm^−1^.

## 3. Methods and Materials

### 3.1. Cell Cultures

Caco-2 cells (2 × 10^4^) were grown at 37 °C and 5% CO_2_ in Dulbecco’s Modified Eagle’s Medium, consisting of 10% FBS, 0.5% glutamine (20 mM, Gibco, Scotland, UK) and 0.5% penicillin (1 mg/100mL), Gibco, Scotland, UK). For making subcultures, the cells were separated from the flask using trypsin/EDTA (Gibco, UK), which was then arrested using the media culture liquid with 10% FBS. Centrifugation occurred at 120× *g*/5 min. 

### 3.2. Sample Preparation

Cells were grown in 6-well plates until they were 80% confluent, then and incubated at 37 °C/24 h with lipids (100 μg/mL fish oil or ML) that had been UV-radiated for 24, 48 or 72 h. The cells were washed twice with PBS, scraped and centrifuged for 5 min at 300× *g* at 4 °C. The supernatant was removed and the pellet was stored at −80 °C until analysis.

### 3.3. ESR Spectroscopy Measurements

Collected cells were mixed with spin trap 2-methyl-2-nitrosopropane (MNP) in DMSO. This mixture was kept at −20 °C for one week before ESR measurement. ESR spectra were verified in a quartz tube using Jeol X-band spectrometer (Hmb, Germany), which operated at 9.71 GHz using a 100 kHz modulating magnetic field. First-derivative spectra were noted, developed into a PC by EW software (Scientific Services), then processed by Excel [34]. 

### 3.4. AFM Measurements

Cell suspensions were spin-coated onto pieces of mica and analysed by AFM (NTEGRA, NT-MDT, Moscow, Russia) in 24 h. A silicon cantilever (AC160TN, Olympus) was prepared with a sharp, conical tip and a curvature radius less than 10 nm. The nominal resonant frequency, *fo*, of the cantilever was 280 kHz, and the spring constant, *k*, was 42 N/m. Images were logged concurrently in topographic style and in phase style, the scan sizes ranging from 2 to 50 μm. 

### 3.5. RM Measurements

Since the asymmetry of Raman bands in water molecules can interfere with sample spectra, our samples were freeze-dried. Raman spectra were recorded using a Renishaw System 2000 spectrometer equipped with a Leica microscope for both sample illumination and scattered light collection. A GaAlAs laser (wavelength 782 nm) was used for excitation, producing a maximum of 9 mW at the focal plane of a 50× objective, typically used to illuminate the sample. A grating of 1200 lines per mm was used to disperse the collected and laser-filtered Raman signal across a Peltier cool CCD camera. The spectral resolution was 4 cm^−1^ for all measurements. On-chip integration times ranged up to 128 s, and up to 16 spectra were added to improve the signal-to-noise ratio. The system’s lateral spatial resolution was determined at 1.4 μm using a layered silicon sample, and the depth resolution was 7 μm using a thick polyethylene sample. The RS of every sample was taken as the average of RS from three dissimilar regions of three different experiments.

### 3.6. Statistical Analyses

Data analyses were conducted using SPSS (SPSS Inc., Chicago, IL, USA). One-way ANOVA and Tukey’s post hoc tests were applied using significance at *p* < 0.05. Principal component analysis (PCA) was made for producing principal components (PCs), including a compact number of orthogonal changes which caused the most variance in the spectra. PC is connected to the original spectrum by the PC score, which characterizes a component’s weight against the basic spectrum. In order to analyse the PCA data, a basic demonstration of PC coefficients with included variables was applied [53]. A positive or negative symbol indicates a PC coefficient that is a fourth to a half of the largest value, and a double positive or negative symbol indicates a PC coefficient that was higher than half of the absolute value. An empty space was assigned when the value was less than a fourth of the largest absolute value. 

## 4. Conclusions

This study was the first to investigate the biomolecular modification of cells at the cellular level after exposure to oxidized lipids. Here, cytotoxicity following 24 h exposure of Caco-2 cells to 100 μg/mL oxidized lipids (ML or fish oil) was assessed. Analysis by ESR spectroscopy detected free radicals in the lipid membrane, and RM and AFM revealed changes in chemical composition and morphology, respectively. Such alterations were found to be primarily due to a breakdown of DNA and the cell membrane. Further studies are needed to monitor the conformational changes in DNA by RM and AFM.

## Figures and Tables

**Table 1:** Tentative assignments of important bands in FTIR and Raman spectra in cells of biological specimens [38].

Peak Assignment (cm^−1^)	Assignment ^a^
3059	(C=C-H)(arom) str
2975	CH_3_ str
2935	CH_3_ and CH_2_ str
2870–2890	CH_2_ str
1735	>C=O ester str
1650–1680	Amide I
1614	Tyr
1606	Phe
1575	Guanine, adenine (ring str)
1440–1460	C-H def
1295	CH_2_ def
1230–1295	Amide III
1129	C-N and C-C str
1102	>PO^2−^ str (sym)
1085	C-O str
1061	C-N and C-C str
1004	Phe
852	Tyr (buried)
829	Tyr (exposed)
785	Cytosine, uracil (ring, str)
720	Adenine
665	Guanine
640	Tyr (skeletal)
620	Phe (skeletal)
520–540	S-S str

str = stretching; def = deformation; sym = symmetric; asym = antisymmetric.

**Table 2:** Relative intensity values at selected regions of Raman spectra for Caco-2 cells treated with UV-radiated ML or fish oil (F). Data is expressed as mean ± SD for three baseline-corrected and normalized spectra of 128 co-added scans.

Assignment ^a^	Wave Number (±2 cm^−1^)	Cont	24 F	48 F	72 F	24 ML	48 ML	72 ML
(C=C-H)(arom) str	3059	0.06 ±0.0001	0.03 ±0.0001	0.03 ±0.0002	0.02 ±0.0002	0.01 ±0.005	0.03 ±0.001	0.013 ±0.001
CH_3_ str	2975	0.22 ±0.006	0.15 ±0.002	0.12 ±0.001	0.14 ±0.001	0.17 ±0.001	0.21 ±0.002	0.13 ±0.001
CH_3_ and CH_2_ str	2935	0.71 ±0.004	0.79 ±0.005	0.31 ±0.001	0.36 ±0.002	0.42 ±0.031	0.61 ±0.004	0.29 ±0.004
CH_2_ str	2878	0.61 ±0.002	0.51 ±0.003	0.25 ±0.002	0.28 ±0.001	0.36 ±0.020	0.48 ±0.005	0.21 ±0.005
>C=O ester str	1735	0.20 ±0.001	0.05 ±0.0001	0.04 ±0.0003	0.01 ±0.0001	0.04 ±0.003	0.07 ±0.0001	0.03 ±0.0001
Amide I	1650	1.1 ±0.023	1.0 ±0.010	0.71 ±0.005	0.67 ±0.012	0.46 ±0.024	0.88 ±0.005	0.68 ±0.005
tyr	1614	0.61 ±0.010	0.25 ±0.001	0.22 ±0.001	0.24 ±0.002	0.18 ±0.021	0.29 ±0.001	0.23 ±0.005
phe	1606	0.48 ±0.024	0.034 ±0.0001	0.17 ±0.001	0.25 ±0.001	0.23 ±0.006	0.30 ±0.002	0.26 ±0.001
Guanine, adenine (ring str)	1575	0.30 ±0.002	0.21 ±0.005	0.19 ±0.002	0.11 ±0.001	0.16 ±0.001	0.13 ±0.010	0.24 ±0.002
CH_2_ def	1295	0.99 ±0.023	0.62 ±0.002	0.5 ±0.003	0.43 ±0.001	0.60 ±0.002	0.59 ±0.003	0.64 ±0.003
Amide III	1254	0.58 ±0.007	0.53 ±0.002	0.44 ±0.006	0.40 ±0.001	0.41 ±0.001	0.49 ±0.006	0.32 ±0.002
C-N and C-C str	1129	0.03 ±0.001	0.14 ±0.001	0.21 ±0.002	0.24 ±0.005	0.26 ±0.003	0.26 ±0.001	0.23 ±0.003
>PO^2−^ str (sym)	1102	0.31 ±0.002	0.49 ±0.005	0.32 ±0.001	0.30 ±0.001	0.29 ±0.005	0.35 ±0.024	0.35 ±0.005
C-O str	1085	0.47 ±0.005	0.58 ±0.024	0.29 ±0.003	0.32 ±0.001	0.32 ±0.002	0.36 ±0.002	0.32 ±0.001
C-N and C-C str	1061	0.48 ±0.006	0.48 ±0.003	0.22 ±0.002	0.21 ±0.003	0.19 ±0.010	0.20 ±0.001	0.19 ±0.002
phe	1004	0.66 ±0.020	0.54 ±0.005	0.48 ±0.006	0.39 ±0.002	0.12 ±0.001	0.42 ±0.010	0.22 ±0.002
Tyr (buried)	852	0.13 ±0.002	0.13 ±0.001	0.16 ±0.001	0.15 ±0.002	0.20 ±0.011	0.23 ±0.005	0.15 ±0.001
Tyr (exposed)	829	0.26 ±0.004	0.47 ±0.001	0.13 ±0.002	0.12 ±0.001	0.14 ±0.003	0.17 ±0.001	0.11 ±0.001
Cytosine, Uracil (ring, str)	785	0.27 ±0.002	0.21 ±0.007	0.1 ±0.006	0.07 ±0.001	0.1 ±0.001	0.12 ±0.003	0.08 ±0.001
Adenine	720	0.44 ±0.004	0.2 ±0.001	0.17 ±0.001	0.19 ±0.003	0.11 ±0.002	0.27 ±0.001	0.05 ±0.001
Guanine	665	0.18 ±0.001	0.09 ±0.002	0.08 ±0.002	0.05 ±0.005	0.04 ±0.001	0.09 ±0.002	0.05 ±0.001
Tyr (skeletal)	640	0.51 ±0.002	0.37 ±0.004	0.27 ±0.002	0.10 ±0.001	0.09 ±0.003	0.14 ±0.005	0.08 ±0.005
Phe (skeletal)	620	0.14 ±0.0005	0.11 ±0.002	0.09 ±0.001	0.07 ±0.0001	0.06 ±0.000	0.08 ±0.001	0.06 ±0.003
S-S str	520	0.23 ±0.001	0.15 ±0.002	0.09 ±0.0001	0.08 ±0.0002	0.09 ±0.001	0.14 ±0.001	0.15 ±0.001

**Table 3:** Ratio of the peak area of the 1302 cm^−1^ band assigned to the lipid deformation mode, or at 1336 cm^−1^ assigned to DNA bases guanine and adenine and the area of amide III bands related to protein vibration. Caco-2 cells were treated with 24, 48 and 72 h UV-radiated fish oil (F) or methyl linoleate (ML).

Sample Caco-2 Cells	Ratio (1302 cm^−1^/Amide III)	Ratio (1336 cm^−1^/Amide III)
Untreated	1.7 ± 0.002	2.1 ± 0.001
24 h (F)	1.4 ± 0.001	1.5 ± 0.001
48 h (F)	1.3 ± 0.001	1.3 ± 0.003
72 h (F)	1.2 ± 0.003	1.1 ± 0.001
24 h (ML)	1.2 ± 0.001	0.6 ± 0.002
48 h (ML)	1.4 ± 0.001	1.3 ± 0.001
72 h (ML)	1.3 ± 0.002	0.7 ± 0.0001

**Table 4:** PC solutions of the FT-Raman spectra data of untreated and treated Caco-2 cells with oxidized lipids.

PC	Eigenvalues	% of Variance	Cumulative %
1	15.882	66.175	66.175
2	4.342	18.091	84.266

**Table 5:** PC coefficients for the relevant FT-Raman microscopy spectral data from PC1 and PC2. A greater difference from 0 indicates a greater contribution of that peak to the PCA separation for Caco-2 cells treated with oxidized lipids.

Wave Number (cm^−1^)	Component	
	1	2
520	++	
620	++	
640	++	
665	++	+
720	++	+
785	++	
829	++	--
852	-	+
1004	++	
1061	++	-
1085	++	--
1102	+	--
1129	--	
1254	++	
1295	++	+
1575	++	++
1606	+	++
1614	++	+
1650	++	
1735	++	+
2878	++	
2935	++	-
2975	++	+
3059	++	

## Abbreviations

AFM	Atomic force microscopy
ESR	Electron spin resonance spectroscopy
F	Fish oil
FTIR	Fourier-transform infrared spectroscopy
ML	Methyl linoleate
PCA	Principal component analysis
PUFAs	Polyunsaturated fatty acids
PV	Peroxide value
RM	Raman microscopy
ROS	Reactive oxygen species
